# Pimecrolimus Is a Potent Inhibitor of Allergic Reactions to Hymenopteran Venom Extracts and Birch Pollen Allergen *In Vitro*

**DOI:** 10.1371/journal.pone.0142953

**Published:** 2015-11-12

**Authors:** Petr Heneberg, Kamila Riegerová, Petr Kučera

**Affiliations:** 1 2nd Department of Internal Medicine, Third Faculty of Medicine, Charles University in Prague, Prague, Czech Republic; 2 Department of Immunology, Third Faculty of Medicine, Charles University in Prague, Prague, Czech Republic; Cincinnati Children's Hospital Medical Center, University of Cincinnati College of Medicine, UNITED STATES

## Abstract

Pimecrolimus (Elidel, SDZ ASM 981) is an anti-inflammatory and immunomodulatory 33-epichloro-derivative of macrolactam ascomycin, with low potential for affecting systemic immune responses compared with other calcineurin inhibitors, cyclosporin A and tacrolimus. Despite numerous studies focused on the mechanism of pimecrolimus action on mast cells, only the single report has addressed pimecrolimus effects on other typical FcεRI-expressing cells, the basophils. Patients allergic to birch pollen (n = 20), hymenopteran venoms (n = 23) and 10 non-allergic volunteers were examined. Primary human basophils pre-treated or not with 0.5–50 μMol pimecrolimus were exposed to various concentrations of recombinant Bet v 1a allergen, bee or wasp venom extracts and anti-IgE for 20 min, and then examined for the expression of CD45, CD193, CD203c, CD63 and CD164 using flow cytometry. The externalization of basophil activation markers (CD63 and CD164) was equally inhibited through pimecrolimus in cells activated by recombinant pollen allergen, hymenopteran venom extracts and anti-IgE. Although the individual response rate was subject to strong variation, importantly, pre-treatment with pimecrolimus lowered the number of activated basophils in response to any of the stimuli in the basophils from all patients. The inhibition was concentration-dependent; approximately half of the basophils were inhibited in the presence of 2.5 mMol pimecrolimus. Pimecrolimus is a valuable new tool for the inhibition of hyper-reactive basophils in patients with pollen allergy and a history of anaphylactic reactions to bee or wasp venoms. Further research should address short-term use of pimecrolimus *in vivo* in a wide spectrum of allergic diseases.

## Introduction

Over the last three decades, the rates of asthma and allergic diseases have increased worldwide, with over half of the U.S. population aged 6 to 59 years sensitive to one or more allergens [1]. In the U.K., treatment of allergies costs more than one billion pounds annually, requires 183,000+ bed-days [2], and accounts for 11% of total primary care prescribing costs [2–3]. In Northern, Central and Eastern Europe, the most allergenic tree pollen is produced by birch (*Betula*) [4–5], inducing locally highly prevalent pollen allergies, with a prevalence of up to 54% of the general population, such as in Zurich [4]. Systemic allergic reactions to bee or wasp (hymenopteran) venom occur in 0.3 to 7.5% of adults across multiple populations [6–10], but 15 to 25% of the general population can be sensitized as shown for rural populations and bee keepers [6]. The frequency of severe anaphylaxis to venom, drug and food allergens is 1–3:10,000 persons, and is lethal in 0.65 to 2% of patients [11].

Pimecrolimus (Elidel, SDZ ASM 981) is an anti-inflammatory and immunomodulatory 33-epichloro-derivative of the macrolactam ascomycin. Pimecrolimus blocks the activation of mast cells and T lymphocytes [12] but not dendritic cells [13]. Pimecrolimus cream is in clinical use as an alternative to corticosteroids to treat skin inflammation, particularly for a short-term and non-continuous chronic treatment of mild to moderate atopic dermatitis in sensitive skin areas of non-immunocompromised patients non-responding to other local therapy; off-label use includes allergic contact dermatitis, irritant contact dermatitis and vitiligo [14]. Similarly to tacrolimus and cyclosporin A, pimecrolimus suppresses histamine release through calcineurin inhibition, preventing NFAT dephosphorylation, cytokines production and degranulation. Pimecrolimus does not affect complement C5a receptor and G-proteins. Some authors have argued that calcineurin inhibitors rather inhibit antibody production instead of directly affecting cutaneous mast cells or basophils [15]. The effects of pimecrolimus can be antagonized by rapamycin, which competitively binds to macrophilin but does not itself affect the cell activation stimulated by FcεRI crosslinking [16]. In contrast to other calcineurin inhibitors, pimecrolimus has negligible effects on systemic immune responses [17–18].

Although numerous studies have addressed the mechanism of pimecrolimus action on mast cells, only a single report addressed pimecrolimus effects on other characteristic FcεRI-expressing cells, the basophils [19]. Our study is the first to address the effects of pimecrolimus on the activation of primary human basophils by naturally occurring stimuli, such as birch pollen allergen, and bee or wasp venom extracts, revealed using the basophil activation test (BAT) and compared to the anti-IgE treatment.

## Materials and Methods

### Study population

Three independent cohorts of adult (23–73 years) human subjects of the Czech nationality were recruited for the BAT tests after written informed consent. Both, the informed consent form and the experiments, were approved by the Ethics Committee of the Third Faculty of Medicine, Charles University in Prague; the approval was not numbered and was dated 25-Jun-2012. The absolute exclusion criteria included the use of antihistamines or oral steroids within 4 days prior to the challenge. The following cohorts were screened:

1. Subjects reporting subjective birch pollen allergy symptoms, with records of at least one positive skin prick test (≥3 mm) for birch pollen allergen or elevated IgE specific to birch pollen allergen (n = 20).
2. Subjects with the history of systemic anaphylactic reactions to hymenopteran (bee or wasp) venom of grade II to IV as classified according to the H. Mueller schedule, positive skin test results and/or elevated specific IgE levels (n = 23).
3. Control subjects without any known history of asthma, allergy and anaphylaxis (n = 10).

We did not apply any relative exclusion criteria based on the ratios of cells positive for basophil activation markers. Only the blood specimens with negative results in anti-IgE stimulations were excluded from the presentation and statistical analyses. For the anti-IgE positive control, the cut-off values were determined by adding the double standard deviation value of CD63^+^CD164^+^ basophils observed in patients`background tubes to the mean value [20]. Three individuals from the cohort of subjects allergic to hymenopteran venom were excluded from the study because they fulfilled the relative exclusion criteria due to insufficient anti-IgE stimulation of their basophils.

### Experimental protocol

**Fig 1:**
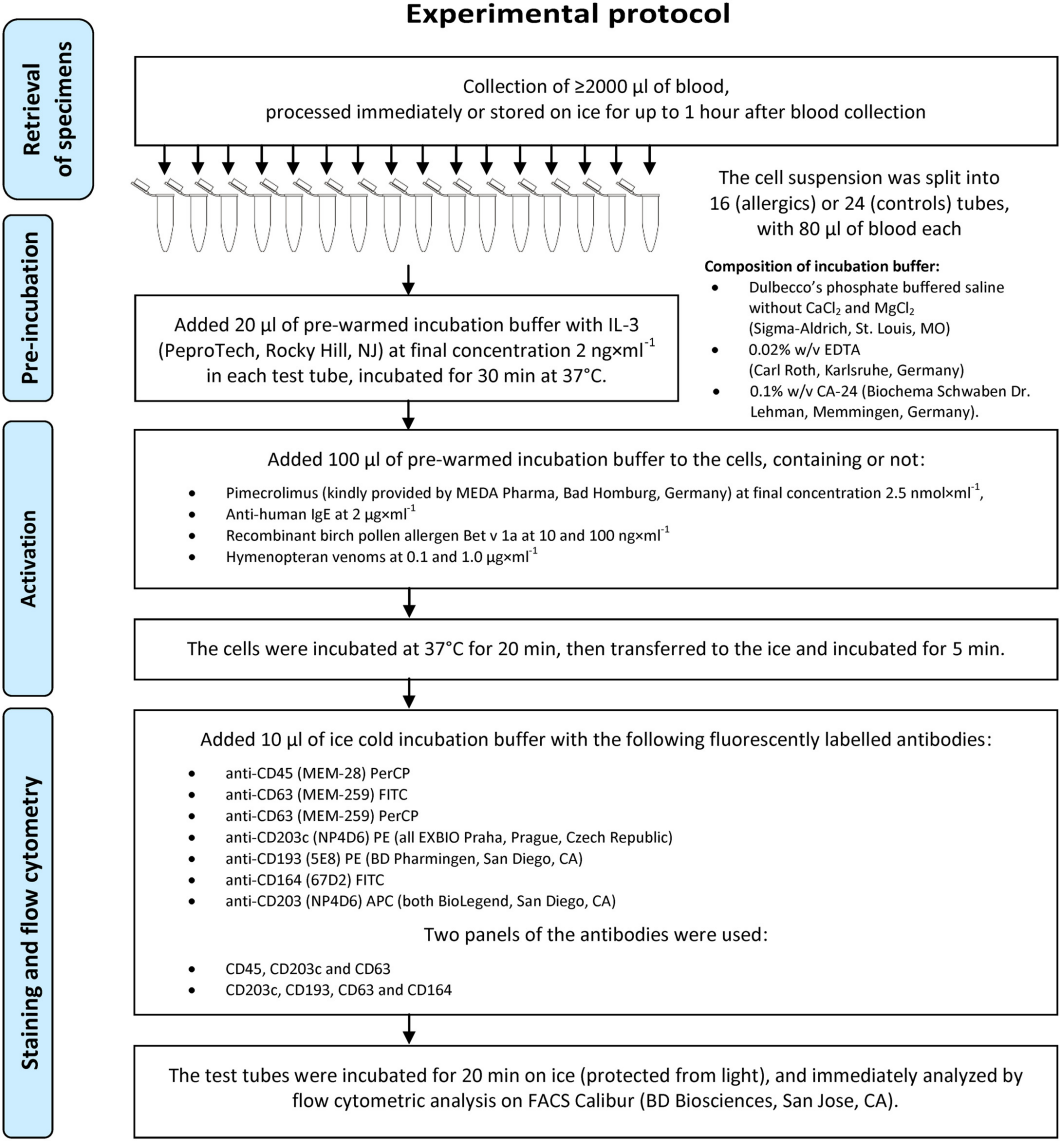
Experimental protocol used for the ex vivo analysis of the pimecrolimus action on basophil activation.

We defined basophils as SSC^low^CD193^+^CD203c^+^ cells. In addition we also quantified the numbers of CD45^dim+high^, CD203c^+^, CD45^dim+high^CD203c^+^ and CD193^+^ cells. Basophil activation was estimated using flow cytometry by measuring increases in CD63 and CD164 on the cell surface prior to and after *ex vivo* stimulation of cells with recombinant birch (*Betula verrucosa*) pollen allergen 1, isoform a (Bet v 1a) (Biomay AG, Vienna, Austria), lyophilized venom extracts of yellow jacket Pharmalgen *Vespula* spp. and honey bee Pharmalgen *Apis mellifera* (both ALK Abelló, Hørsholm, Denmark) and positive control (monoclonal mouse anti-human IgE, clone E124.2.8; Immunotech, Marseille, France). As a negative control, the cells were incubated in an equal volume of the buffer only. A detailed experimental protocol is provided in Fig 1. The pimecrolimus concentration was experimentally optimized using seven subjects, testing doses from 500 nMol to 50 μMol, considering the inhibition of CD63 expression on ~50% of basophils as the target value.

### Data analyses

**Fig 2:**
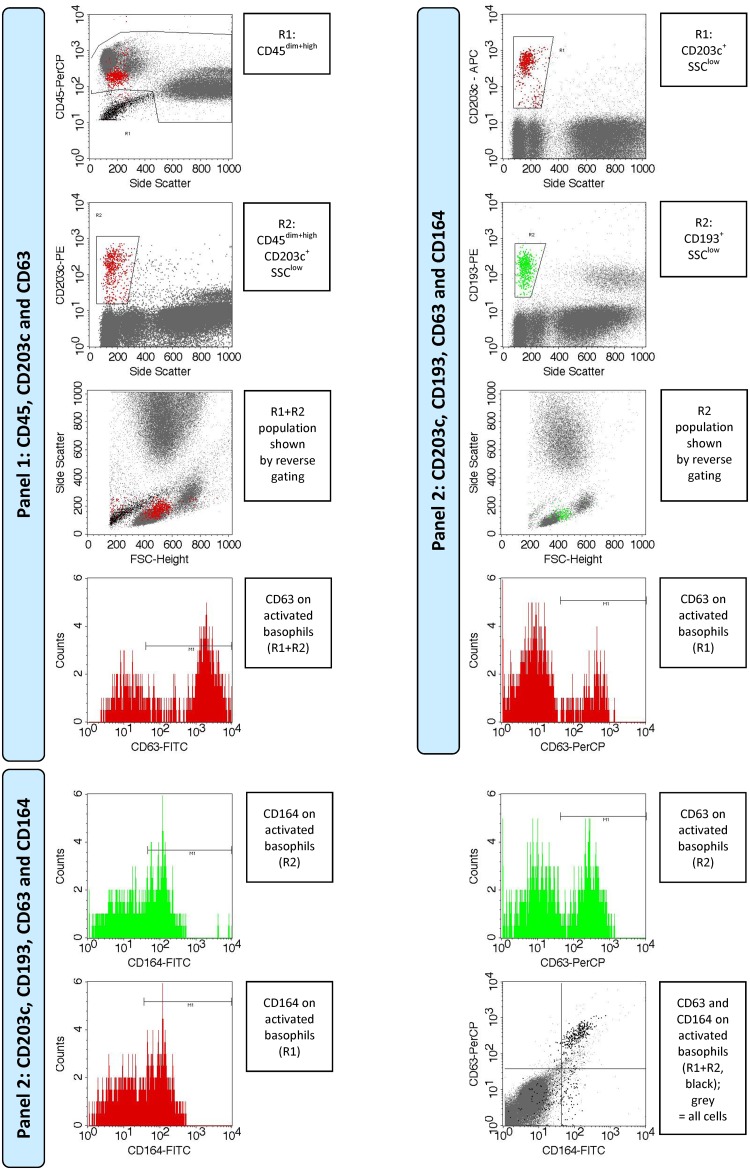
Flowchart diagram of the flow cytometry data analysis.

The data analysis was performed using CellQuest flow cytometry analysis software (BD Biosciences, San Jose, CA) as described in Fig 2. In the first step, the basophil population was gated as SSC^low^CD193^+^CD203c^+^. For the internal control, the following cell populations were gated to ensure that the basophils were correctly identified: SSC^low^CD45^dim+high^, SSC^low^CD203c^+^, SSC^low^CD45^dim+high^CD203c^+^, SSC^low^CD193^+^ and SSC^low^CD203c^+^CD193^+^. In the second step, the percentage of CD63^+^CD164^+^ cells was calculated by comparing the numbers of CD63^+^CD164^+^ cells to the total number of cells expressing the basophil identification markers (Table 1). The cell ratios are shown without subtracting the background. The data are presented as means, standard deviations (SD), and ranges unless stated otherwise. The significance of the obtained data was examined using paired *t*-tests. Bonferroni correction was applied to reduce the chances of obtaining false-positive results (type I errors). The significance of correlations between the pimecrolimus dose and CD63 externalization was analyzed using the linear correlation coefficient r and Spearman’s D.

**Table 1:** Basophil identification and activation markers used in this study.

CD63 (LAMP-3)	Basophil activation marker. Type III lysosomal glycoprotein from the tetraspanin transmembrane family, exposed on the cytoplasmic membrane of human basophils upon activation. It is also present on the surface of activated platelets, monocytes, macrophages, and some non-hematopoietic lineages. In resting basophils, CD63 is limited to the membrane of intracytoplasmatic granules and is barely expressed on the outer cell membrane in both healthy individuals and allergic patients. As a result of the fusion of the granule and the outer cell membrane, CD63 is exposed at high density on IgE- or allergen-activated cells [21].
CD164 (MUC-24)	Basophil activation marker. Heavily N- and O-glycosylated transmembrane sialomucin exposed on the cytoplasmic membrane of human basophils upon activation. It is also present on the surface of other cells of the hematopoietic lineage, including the CD34^+^ myeloid and erythroid progenitors, on some non-hematopoietic lineages and transformed cells [22].
CD45 (LCA)	Type I membrane glycoprotein considered a marker of hematopoietic cells, except erythrocytes and platelets.
CD203c (E-NPP3)	Basophil identification marker. Transmembrane ectoenzyme with ATPase and ATP pyrophosphatase activity, expressed on basophils and mast cells (at relatively low levels in some resting basophils and upregulated upon activation). It is also expressed on some non-hematopoietic cell types [23, 24].
CD193 (CCR3)	Basophil identification marker. G protein-coupled seven transmembrane receptor for eotaxins, RANTES and MCPs. CD193 also serves also as a co-receptor for HIV-1 and HIV-2. CD193 is expressed on human basophils, eosinophils, mast cells, mononuclear cells, platelets, T_H_2-like lymphocytes, hematopoietic progenitors and keratinocytes.

## Results

### Optimization of pimecrolimus concentrations

**Fig 3:**
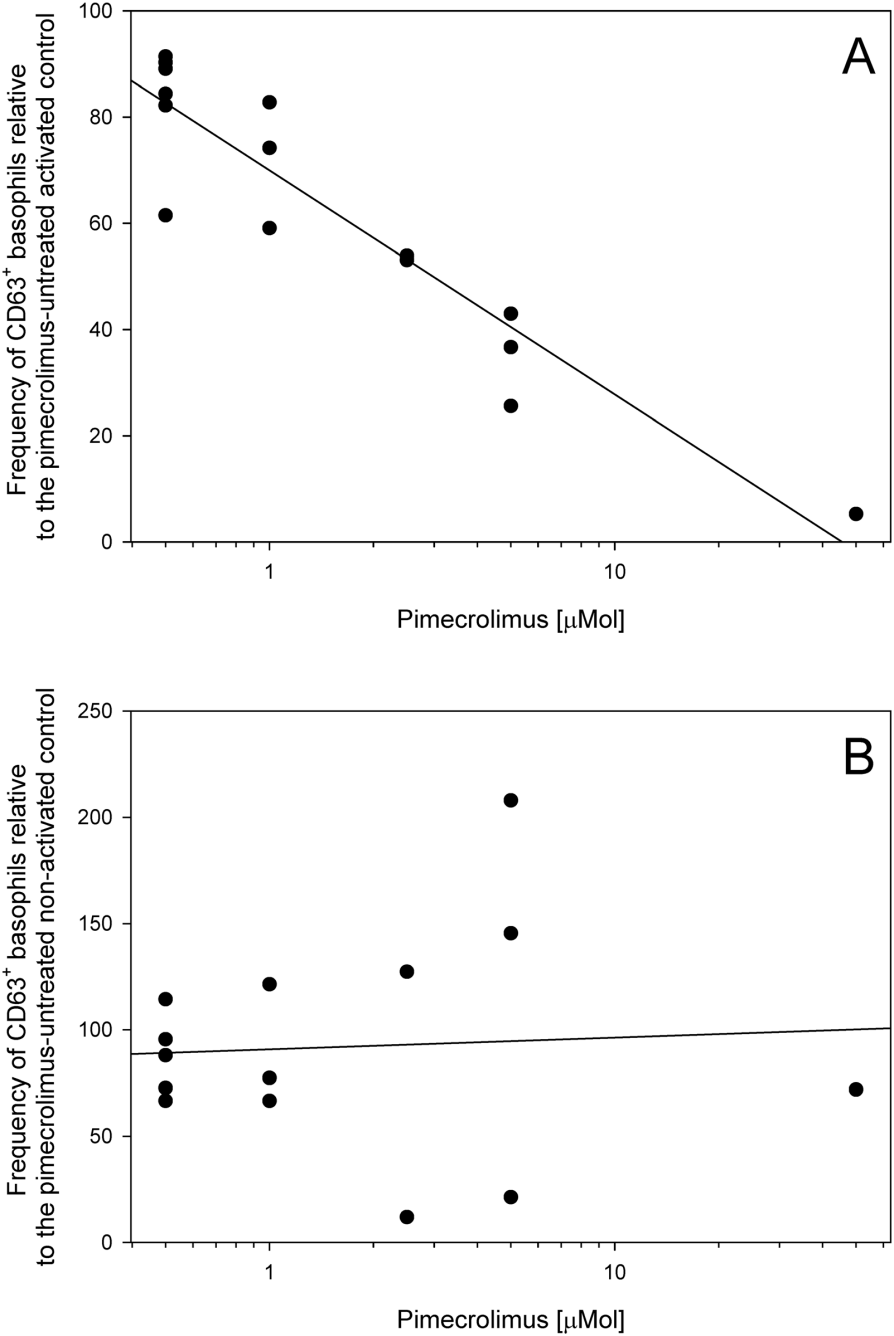
Inhibition of CD63 externalization on the surface of primary human basophils activated (A) or not (B) following treatment with 0.5–50 μMol pimecrolimus. (**A**) Basophils pre-treated with various concentrations of pimecrolimus were activated for 20 min with anti-IgE (n = 1 subject), bee venom (n = 3 subjects) or wasp venom (n = 3 subjects). Linear correlation coefficient r = -0.696, p < 0.01; Spearman’s D = 1027, p < 0.01. (**B**) The basophils were only pre-treated with pimecrolimus but were not activated by any external stimulus. Linear correlation coefficient r = -0.074, p > 0.05; Spearman’s D = 514, p > 0.05.

Preincubation of primary human basophils with pimecrolimus resulted in a dose-dependent inhibition of basophil activation, revealed as down-regulated CD63 externalization. The inhibition of CD63 externalization on ~50% of basophils was obtained using 2.5 μMol pimecrolimus (Fig 3); this concentration was thus used throughout the remainder of the study.

### Non-stimulated basophils

**Fig 4:**
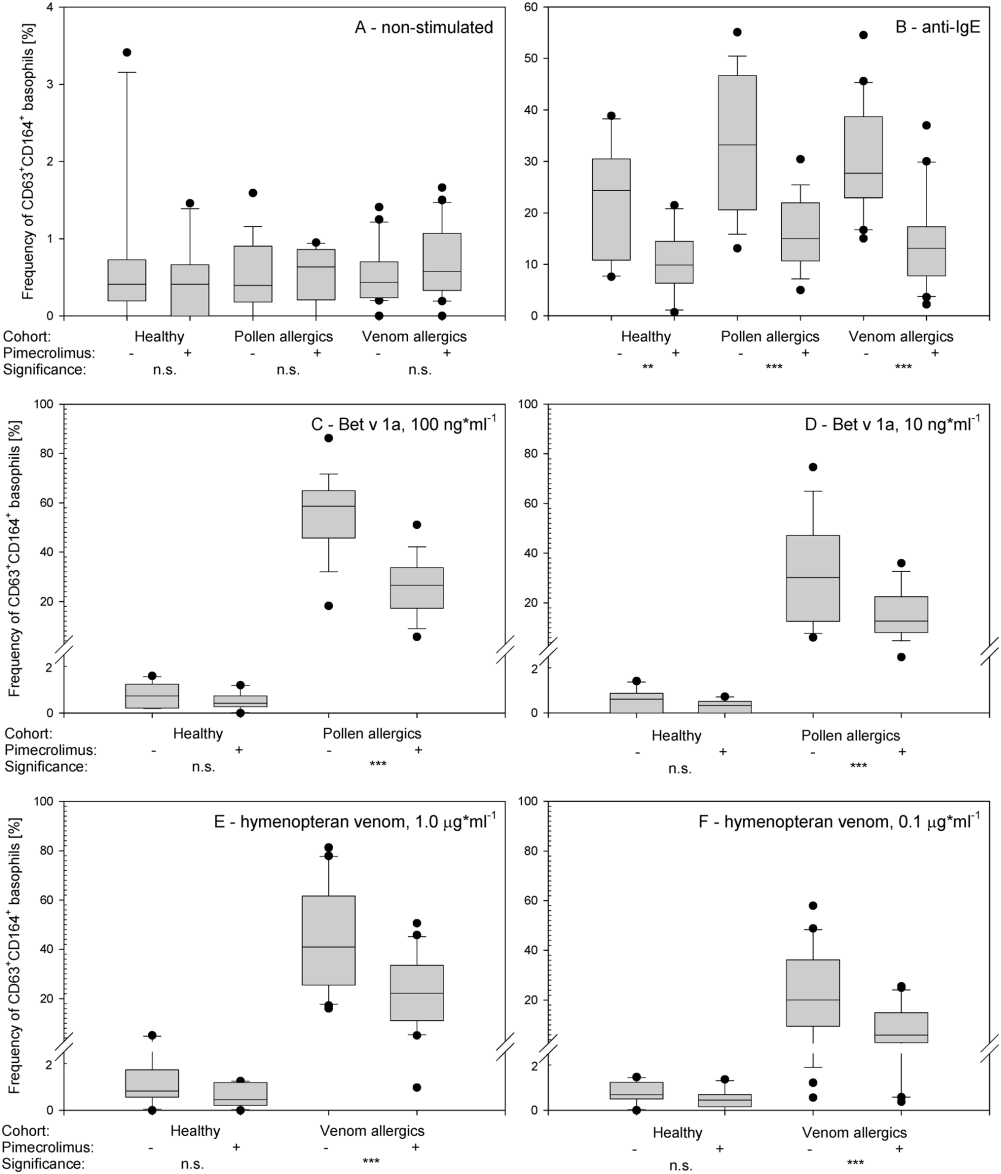
Effects of pimecrolimus treatment on the externalization of CD63 and CD164. The frequency [%] of CD63^+^CD164^+^ cells among the total basophils (gated as SSC^low^CD193^+^CD203c^+^) is shown. The IL-3-primed basophils were pre-treated or not with 2.5 μMol pimecrolimus and analyzed prior to (**A**) and after activation with anti-IgE (**B**), birch pollen allergen Bet v 1a at 100 ng*ml^-1^ (**C**) and 10 ng*ml^-1^ (**D**), and hymenopteran venom at 1.0 μg*ml^-1^ (**E**) and 0.1 μg*ml^-1^ (**F**). The Tukey box plots are shown, the mean values are presented as lines, and the 5^th^ and 95^th^ percentiles are displayed as symbols. The significance was examined by the paired t-tests with Bonferroni correction at n = 18 and is indicated as *** (p_corr_<0.001 = p_uncorr_<0.000056), ** (p_corr_<0.01 = p_uncorr_<0.00056), * (p_corr_<0.05 = p_uncorr_<0.0028) and n.s. (p_corr_>0.05 = p_uncorr_<0.0028).

Pre-treatment of non-stimulated and IL-3-primed basophils with 2.5 μMol pimecrolimus did not affect the expression of activation markers. The SSC^low^CD193^+^CD203c^+^CD63^+^CD164^+^ cells were nearly absent both prior to and after the incubation with pimecrolimus in leukocytes obtained from healthy volunteers (Fig 4; paired *t*-test *p*>0.05).

### Stimulation with anti-IgE

**Fig 5:**
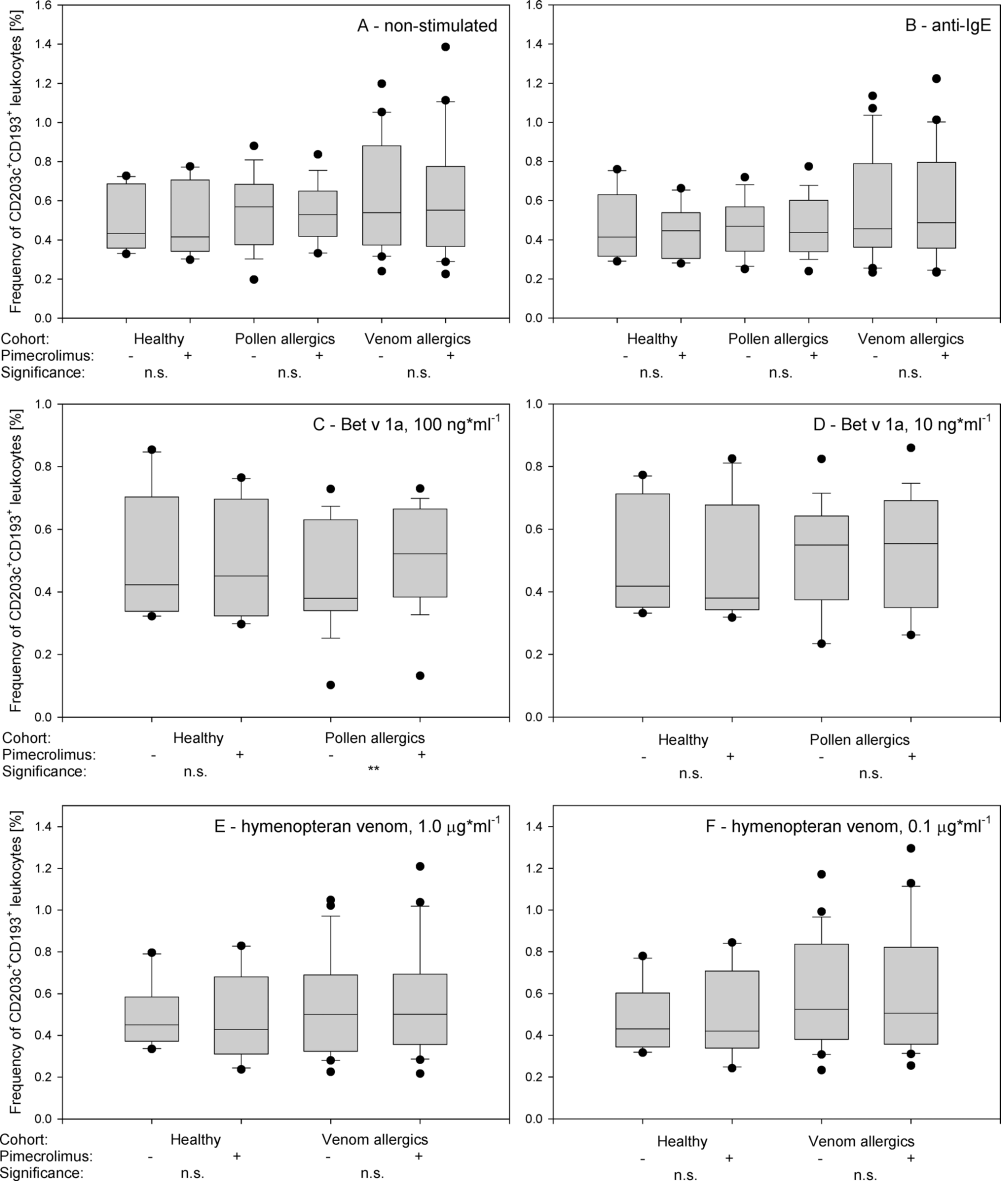
Effects of pimecrolimus treatment on the externalization of CD193 and CD203c. The frequency [%] of SSC^low^CD203c^+^CD193^+^ cells among the total leukocytes is shown. The IL-3-primed basophils were pre-treated or not with 2.5 μMol pimecrolimus and analyzed prior to (**A**) and after activation with anti-IgE (**B**), birch pollen allergen Bet v 1a at 100 ng*ml^-1^ (**C**) and 10 ng*ml^-1^ (**D**), and hymenopteran venom at 1.0 μg*ml^-1^ (**E**) and 0.1 μg*ml^-1^ (**F**). The Tukey box plots are shown, the mean values are presented as lines, and the 5^th^ and 95^th^ percentiles are displayed as symbols. The significance was examined by the paired t-tests with Bonferroni correction at n = 18 and is indicated as *** (p_corr_<0.001 = p_uncorr_<0.000056), ** (p_corr_<0.01 = p_uncorr_<0.00056), * (p_corr_<0.05 = p_uncorr_<0.0028) and n.s. (p_corr_>0.05 = p_uncorr_<0.0028).

Pimecrolimus inhibited the activation of basophils stimulated with anti-IgE. The total number of basophils (SSC^low^CD203c^+^CD193^+^, or defined by other combinations of markers) activated with anti-IgE did not change (Fig 5, S1–S3 Figs), but the basophil activation markers were significantly down-regulated in response to pre-incubation with pimecrolimus (paired *t*-test *p*<0.001 for each combination; Fig 4B, S4–S8 Figs). In response to pimecrolimus treatment, the frequency of anti-IgE-activated CD63^+^CD164^+^ basophils decreased from 22.0±10.8% to 10.3±6.1% in healthy subjects, from 33.1±13.5% to 16.0±6.7% in pollen allergics, and from 29.9±10.5% to 14.8±9.5% in venom allergics. The extent of the pimecrolimus-induced response to anti-IgE treatment was similar for all three cohorts (decrease of CD63^+^CD164^+^ basophils by 53.0%, 51.4% and 50.6% in controls, pollen and venom allergics, respectively).

### Stimulation with birch pollen allergen

Pimecrolimus inhibited the activation of basophils stimulated with the recombinant birch pollen antigen Bet v 1a. Pimecrolimus induced a significant increase in the externalization of both CD203c and CD193 on the surface of cells isolated from birch pollen allergics (but not healthy controls) following stimulation with high doses of allergen (100 ng*ml^-1^), leading to a 10–14% increase in the frequency of SSC^low^CD203c^+^CD193^+^, SSC^low^CD203c^+^ and SSC^low^CD193^+^ cells (paired *t*-test *p*<0.001 for each combination). A similar trend was detected in cells from birch pollen allergics stimulated with low doses of allergen (10 ng*ml^-1^; decrease by 2–5%, paired *t*-test *p* = 0.05, >0.05, <0.01, respectively), but was absent in cells isolated from healthy subjects stimulated with pollen allergen at both doses tested (Fig 5C and 5D, S1–S3 Figs).

Basophil activation markers were significantly down-regulated in pollen allergen-activated cells in response to pre-incubation with pimecrolimus (paired *t*-test *p* < 0.001 for each combination; Fig 4C and 4D, S4–S8 Figs). In response to pimecrolimus treatment, the frequency of CD63^+^CD164^+^ basophils stimulated with high-dose pollen allergen decreased from 54.9±16.1% to 26.1±11.7% in birch pollen allergics, and from 0.8±0.5% to 0.5±0.4% in healthy subjects, and the frequency of CD63^+^CD164^+^ basophils stimulated with low-dose pollen allergen decreased from 33.3±19.8% to 15.5±9.6% in birch pollen allergics, and from 0.5±0.5% to 0.3±0.3% in healthy subjects. The extent of the pimecrolimus-induced response in birch pollen allergics was similar after treatment with both doses of pollen allergen tested (52.4% and 53.5% decrease of CD63^+^CD164^+^ basophils, respectively).

### Stimulation with hymenopteran venom extract

Pimecrolimus inhibited the activation of basophils stimulated with yellow jacket and honey bee venom extracts. Pimecrolimus treatment increased the externalization of both CD203c and CD193 on the surface of cells isolated from hymenopteran venom allergics (but not healthy controls) following stimulation with high doses of venom (1.0 μg*ml^-1^), leading to a 4.3–6.6% increase in the frequency of SSC^low^CD203c^+^CD193^+^, SSC^low^CD203c^+^ and SSC^low^CD193^+^ cells (borderline significance—paired *t*-test *p* = 0.02, = 0.08 and = 0.02, respectively). We did not observe a similar trend using lower doses of the venom (0.1 μg*ml^-1^; paired *t*-test *p* > 0.05 each), and this effect was also absent in cells isolated from healthy subjects stimulated with hymenopteran venom at any dose (Fig 5E and 5F, S1–S3 Figs).

Basophil activation markers were significantly down-regulated in hymenopteran venom-activated cells in response to pre-incubation with pimecrolimus (paired *t*-test *p* < 0.001 for each combination; Fig 4E and 4F, S4–S8 Figs). In response to pimecrolimus treatment, the frequency of CD63^+^CD164^+^ basophils stimulated with high-dose hymenopteran venom decreased from 44.4±20.7% to 22.9±13.8% in hymenoptera venom allergics, and from 1.3±1.4% to 0.6±0.5% in healthy subjects, and the frequency of CD63^+^CD164^+^ basophils stimulated with low-dose hymenopteran venom decreased from 22.8±16.5% to 8.3±7.8% in hymenoptera venom allergics, and from 0.8±0.5% to 0.5±0.4% in healthy subjects. The extent of pimecrolimus-induced response to hymenopteran venom extract treatment of hymenopteran venom allergics was similar after treatment with both, high and low doses of hymenopteran venom extract (48.5% and 63.5% decrease of CD63^+^CD164^+^ basophils, respectively).

## Discussion

Calcineurin inhibitors are monitored by FDA. Following topical pimecrolimus treatment, there were reported 10 postmarketing cancer cases: skin cancers, lymphomas and granulomatous lymphadenitis [25], suggesting photocarcinogenicity as a causative factor. However, three independent studies showed no difference between the pimecrolimus-treated and control mice treated with UV or simulated solar radiation [26–28]. In addition, the risk of lymphoma following exposure to topical calcineurin inhibitors was not increased in atopic dermatitis patients [29]. Thus, a decade ago, EMEA has argued that the available data on possible risk of carcinogenesis are inconclusive, and requested further long-term safety profiles to provide a definitive answer. Recent consensus recommendations conclude that these restrictions are no longer justified and recommend that regulatory authorities should remove the respective passages [30].

Current therapeutic approaches in allergy and anaphylaxis prefer to target basophil mediators, considering histamine antagonists as the most commonly used compounds. Alternative approaches are represented by the direct inhibition of basophil activation and the subsequent release of basophil mediators using corticosteroids, systemically administered cyclosporin A, and topically administered tacrolimus. In the present study, we provided the first evidence that pimecrolimus can also be used to inhibit the activation of basophils isolated from patients allergic to birch pollen and patients hypersensitive to bee or wasp venoms. Using the BAT test, we showed that pimecrolimus inhibits the activation of basophils mediated through a broad spectrum of stimuli, including the activation of basophils mediated through recombinant birch pollen allergen, bee or wasp venom extracts, and an artificial anti-IgE stimulus as a positive control. There has only been one previous study concerning the action of pimecrolimus on basophils. Zuberbier *et al*. [19] showed that pimecrolimus induces both histamine and tryptase release in response to anti-IgE treatment. This effect is of particular importance, because tryptase can further amplify the signal through the release of active IL-1ß and IL-8 from human endothelial cells, attracting neutrophils and eosinophils, cleaving C3 to the anaphylatoxin C3a and enhancing bronchial hyperreactivity to histamine [19, 31].

Interestingly, pimecrolimus induced reproducible increases in the number of SSC^low^ white blood cells exposing CD193 and CD203c (including the double-positive SSC^low^CD193^+^CD203c^+^ cells) after stimulation with high doses of allergen or venoms (Fig 5, S1–S3 Figs). Although this observation contradicts the claims of stable CD193 expression on the cell membrane of all basophils [23], and this effect is consistent with the CD193 down-regulation on activated basophils claimed by [32–34]. We did not observe the increased externalization of CD193 and CD203c in pimecrolimus-treated cells activated with low-dose allergen, venom or anti-IgE and non-activated cells (Fig 5, S1–S3 Figs).

At a 2.5 mMol concentration, pimecrolimus down-regulated the exposure of basophil activation markers to approximately half of the levels observed on pimecrolimus-untreated cells activated with any of the stimuli tested. We observed the strongest response to pimecrolimus in cells activated with low-dose hymenopteran venom (63.5% of cells inhibited) and the weakest response in cells activated with high-dose hymenopteran venom (48.5%). The mean response to 2.5 mMol pimecrolimus reached 52.4% and 53.5% in cells treated with high/low dose of birch pollen allergen, respectively, and 50.6–53.0% in cells treated with anti-IgE isolated from the three cohorts tested. Although the individual response rate was subject to strong variation, importantly, pimecrolimus treatment lowered the number of activated basophils in all subjects in response to any of the stimuli tested (without exception).

There is limited information available on the quantification of the inhibition of basophil activation using BAT tests in contrast with the quantification of the activation itself [34]. We are only aware of the analysis of the cyclosporin A-mediated inhibition of CD203c exposure on basophils by Hauswirth *et al*. [35]. Herein, we have shown that pimecrolimus effectively inhibited the membrane exposure of BAT markers in all research subjects, independently of the activator used. The BAT test revealed that the previously reported pimecrolimus-mediated inhibition of basophils [19] is not mediated through the partial inhibition of the response of all the basophils present. The 2.5 mMol pimecrolimus treatment affected the activation of only a part (approximately a half) of the pool of basophils in each research subject, whereas the remaining basophils were still sensitive to the activators. The frequency of pimecrolimus-responding cells was concentration-dependent. The only moderate effects of corticosteroids on type I allergic symptoms, adverse effects of long-term treatment with corticosteroids, and the more pronounced effects of treatment with pimecrolimus compared with dexamethasone and cyclosporin A highlight the potential of pimecrolimus for the treatment of immediate and late-type allergic diseases.

## Supporting Information

S1 Fig Effects of pimecrolimus treatment on the externalization of CD203c. The frequency [%] of SSC^low^CD203c-PE^+^ cells among the total leukocytes is shown. The IL-3-primed basophils were pre-treated or not with 2.5 μMol pimecrolimus and analyzed prior to (**A**) and after activation with anti-IgE (**B**), birch pollen allergen Bet v 1a at 100 ng*ml^-1^ (**C**) and 10 ng*ml^-1^ (**D**), and hymenopteran venom at 1.0 μg*ml^-1^ (**E**) and 0.1 μg*ml^-1^ (**F**). The Tukey box plots are shown, the mean values are presented as lines, and the 5^th^ and 95^th^ percentiles are displayed as symbols.
(PDF)

S2 Fig Effects of pimecrolimus treatment on the externalization of CD203c. The frequency [%] of SSC^low^CD203c-APC^+^ cells among the total leukocytes is shown. The IL-3-primed basophils were pre-treated or not with 2.5 μMol pimecrolimus and analyzed prior to (**A**) and after activation with anti-IgE (**B**), birch pollen allergen Bet v 1a at 100 ng*ml^-1^ (**C**) and 10 ng*ml^-1^ (**D**), and hymenopteran venom at 1.0 μg*ml^-1^ (**E**) and 0.1 μg*ml^-1^ (**F**). The Tukey box plots are shown, the mean values are presented as lines, and the 5^th^ and 95^th^ percentiles are displayed as symbols.
(PDF)

S3 Fig Effects of pimecrolimus treatment on the externalization of CD193. The frequency [%] of SSC^low^CD193-PE^+^ cells among the total leukocytes is shown. The IL-3-primed basophils were pre-treated or not with 2.5 μMol pimecrolimus and analyzed prior to (**A**) and after activation with anti-IgE (**B**), birch pollen allergen Bet v 1a at 100 ng*ml^-1^ (**C**) and 10 ng*ml^-1^ (**D**), and hymenopteran venom at 1.0 μg*ml^-1^ (**E**) and 0.1 μg*ml^-1^ (**F**). The Tukey box plots are shown, the mean values are presented as lines, and the 5^th^ and 95^th^ percentiles are displayed as symbols.
(PDF)

S4 Fig Effects of pimecrolimus treatment on the externalization of CD63. The frequency [%] of CD63-FITC^+^ cells among the total basophils (gated as SSC^low^CD203c-PE^+^) is shown. The IL-3-primed basophils were pre-treated or not with 2.5 μMol pimecrolimus and analyzed prior to (**A**) and after activation with anti-IgE (**B**), birch pollen allergen Bet v 1a at 100 ng*ml^-1^ (**C**) and 10 ng*ml^-1^ (**D**), and hymenopteran venom at 1.0 μg*ml^-1^ (**E**) and 0.1 μg*ml^-1^ (**F**). The Tukey box plots are shown, the mean values are presented as lines, and the 5^th^ and 95^th^ percentiles are displayed as symbols.
(PDF)

S5 Fig Effects of pimecrolimus treatment on the externalization of CD63. The frequency [%] of CD63-PerCP^+^ cells among the total basophils (gated as SSC^low^CD203c-APC^+^) is shown. The IL-3-primed basophils were pre-treated or not with 2.5 μMol pimecrolimus and analyzed prior to (**A**) and after activation with anti-IgE (**B**), birch pollen allergen Bet v 1a at 100 ng*ml^-1^ (**C**) and 10 ng*ml^-1^ (**D**), and hymenopteran venom at 1.0 μg*ml^-1^ (**E**) and 0.1 μg*ml^-1^ (**F**). The Tukey box plots are shown, the mean values are presented as lines, and the 5^th^ and 95^th^ percentiles are displayed as symbols.
(PDF)

S6 Fig Effects of pimecrolimus treatment on the externalization of CD63. The frequency [%] of CD63-PerCP^+^ cells among the total basophils (gated as SSC^low^CD193-PE^+^) is shown. The IL-3-primed basophils were pre-treated or not with 2.5 μMol pimecrolimus and analyzed prior to (**A**) and after activation with anti-IgE (**B**), birch pollen allergen Bet v 1a at 100 ng*ml^-1^ (**C**) and 10 ng*ml^-1^ (**D**), and hymenopteran venom at 1.0 μg*ml^-1^ (**E**) and 0.1 μg*ml^-1^ (**F**). The Tukey box plots are shown, the mean values are presented as lines, and the 5^th^ and 95^th^ percentiles are displayed as symbols.
(PDF)

S7 Fig Effects of pimecrolimus treatment on the externalization of CD164. The frequency [%] of CD164-FITC^+^ cells among the total basophils (gated as SSC^low^CD203c-APC^+^) is shown. The IL-3-primed basophils were pre-treated or not with 2.5 μMol pimecrolimus and analyzed prior to (**A**) and after activation with anti-IgE (**B**), birch pollen allergen Bet v 1a at 100 ng*ml^-1^ (**C**) and 10 ng*ml^-1^ (**D**), and hymenopteran venom at 1.0 μg*ml^-1^ (**E**) and 0.1 μg*ml^-1^ (**F**). The Tukey box plots are shown, the mean values are presented as lines, and the 5^th^ and 95^th^ percentiles are displayed as symbols.
(PDF)

S8 Fig Effects of pimecrolimus treatment on the externalization of CD164. The frequency [%] of CD164-FITC^+^ cells among the total basophils (gated as SSC^low^CD193-PE^+^) is shown. The IL-3-primed basophils were pre-treated or not with 2.5 μMol pimecrolimus and analyzed prior to (**A**) and after activation with anti-IgE (**B**), birch pollen allergen Bet v 1a at 100 ng*ml^-1^ (**C**) and 10 ng*ml^-1^ (**D**), and hymenopteran venom at 1.0 μg*ml^-1^ (**E**) and 0.1 μg*ml^-1^ (**F**). The Tukey box plots are shown, the mean values are presented as lines, and the 5^th^ and 95^th^ percentiles are displayed as symbols.
(PDF)
