# Supplementary figures and images for: Pimecrolimus Is a Potent Inhibitor of Allergic Reactions to Hymenopteran Venom Extracts and Birch Pollen Allergen In Vitro
Source: PLoS One. 2015 Nov 12;10(11):e0142953. doi: 10.1371/journal.pone.0142953 (PMC4643035; doi:10.1371/journal.pone.0142953)

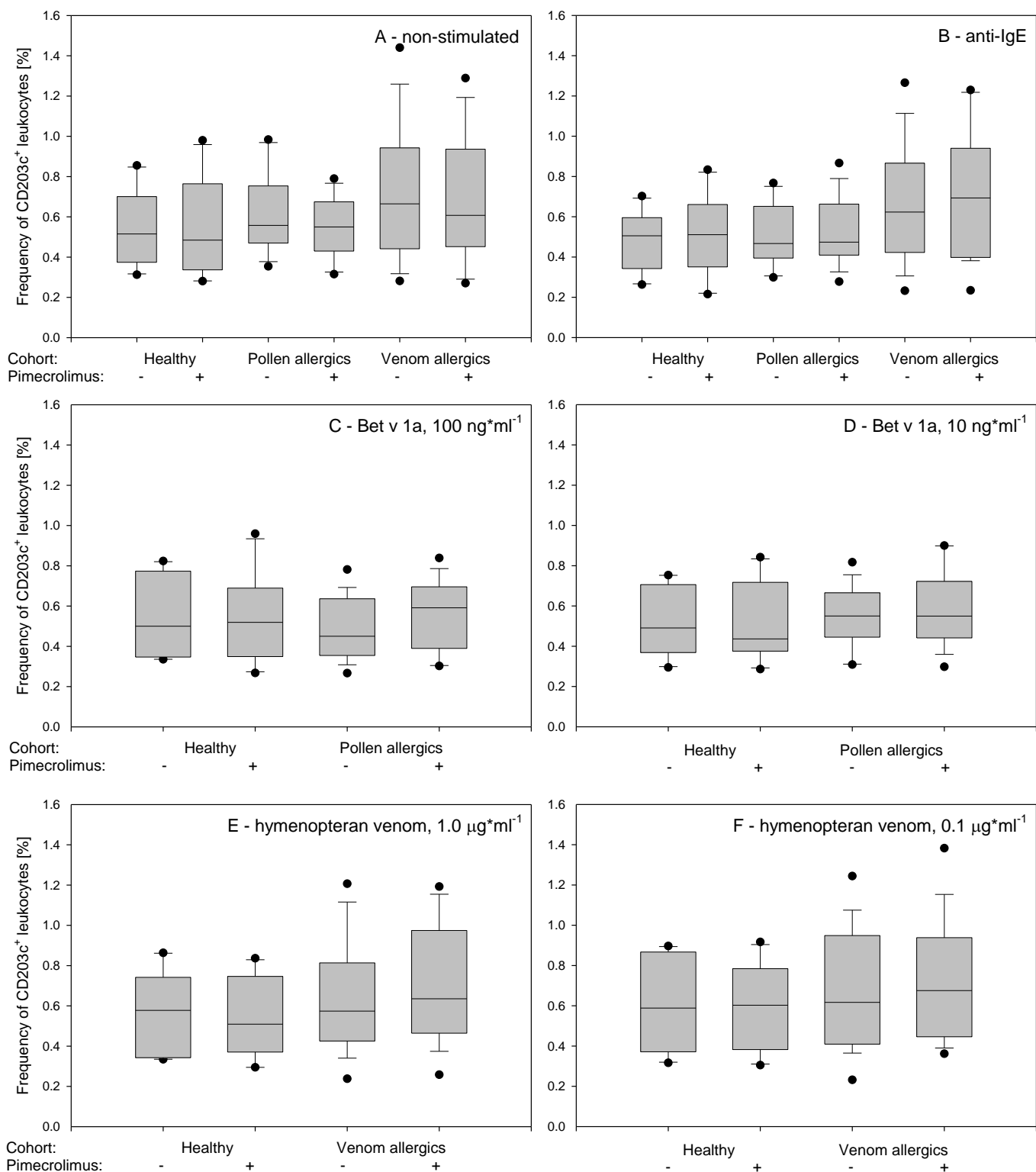

Figure S1

Supplement: S1 Fig — The frequency [%] of SSClowCD203c-PE+ cells among the total leukocytes is shown. The IL-3-primed basophils were pre-treated or not with 2.5 μMol pimecrolimus and analyzed prior to (A) and after activation with anti-IgE (B), birch pollen allergen Bet v 1a at 100 ng*ml-1 (C) and 10 ng*ml-1 (D), and hymenopteran venom at 1.0 μg*ml-1 (E) and 0.1 μg*ml-1 (F). The Tukey box plots are shown, the mean values are presented as lines, and the 5th and 95th percentiles are displayed as symbols. (PDF) [file pone.0142953.s001.pdf]

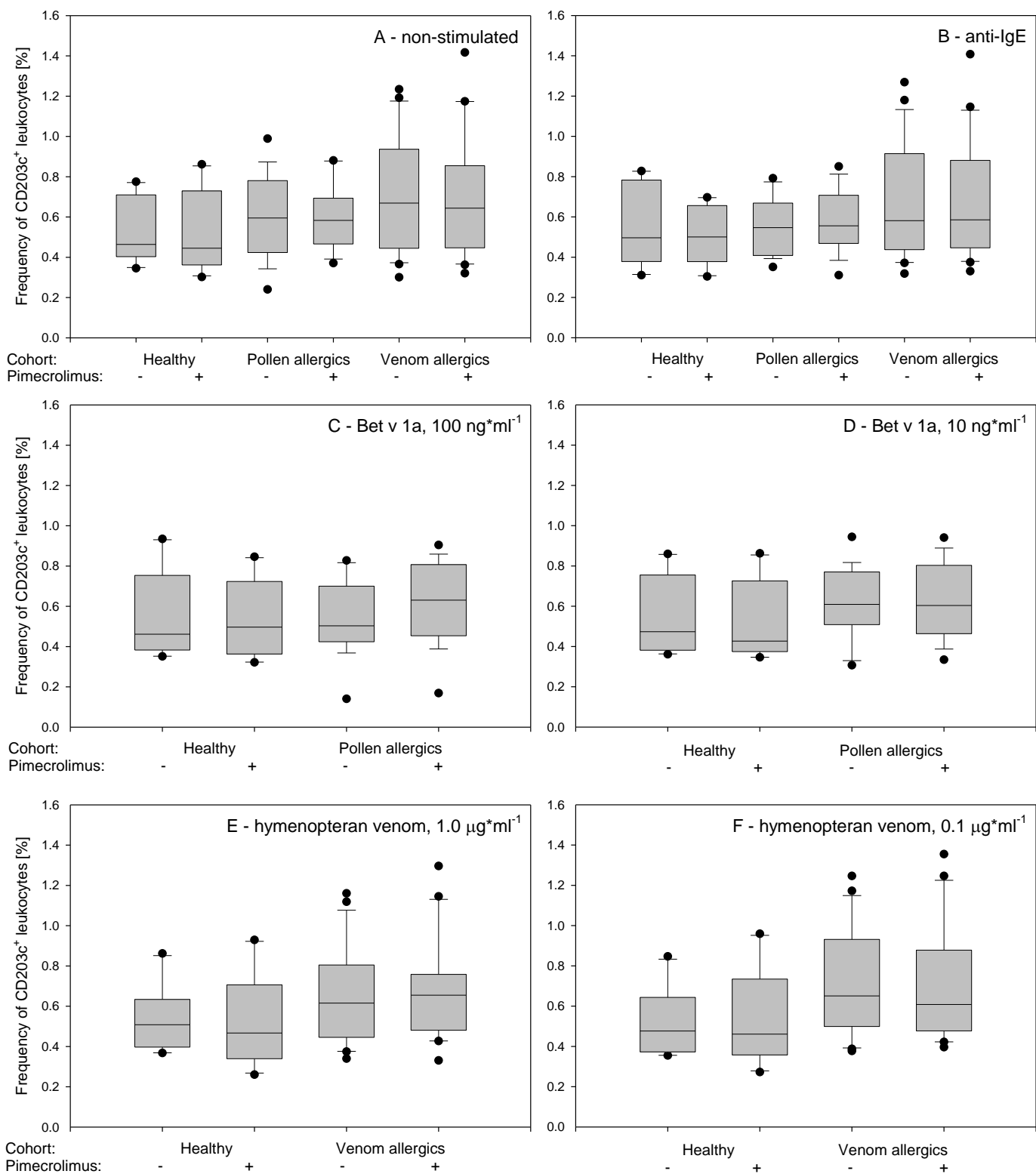

Figure S2

Supplement: S2 Fig — The frequency [%] of SSClowCD203c-APC+ cells among the total leukocytes is shown. The IL-3-primed basophils were pre-treated or not with 2.5 μMol pimecrolimus and analyzed prior to (A) and after activation with anti-IgE (B), birch pollen allergen Bet v 1a at 100 ng*ml-1 (C) and 10 ng*ml-1 (D), and hymenopteran venom at 1.0 μg*ml-1 (E) and 0.1 μg*ml-1 (F). The Tukey box plots are shown, the mean values are presented as lines, and the 5th and 95th percentiles are displayed as symbols. (PDF) [file pone.0142953.s002.pdf]

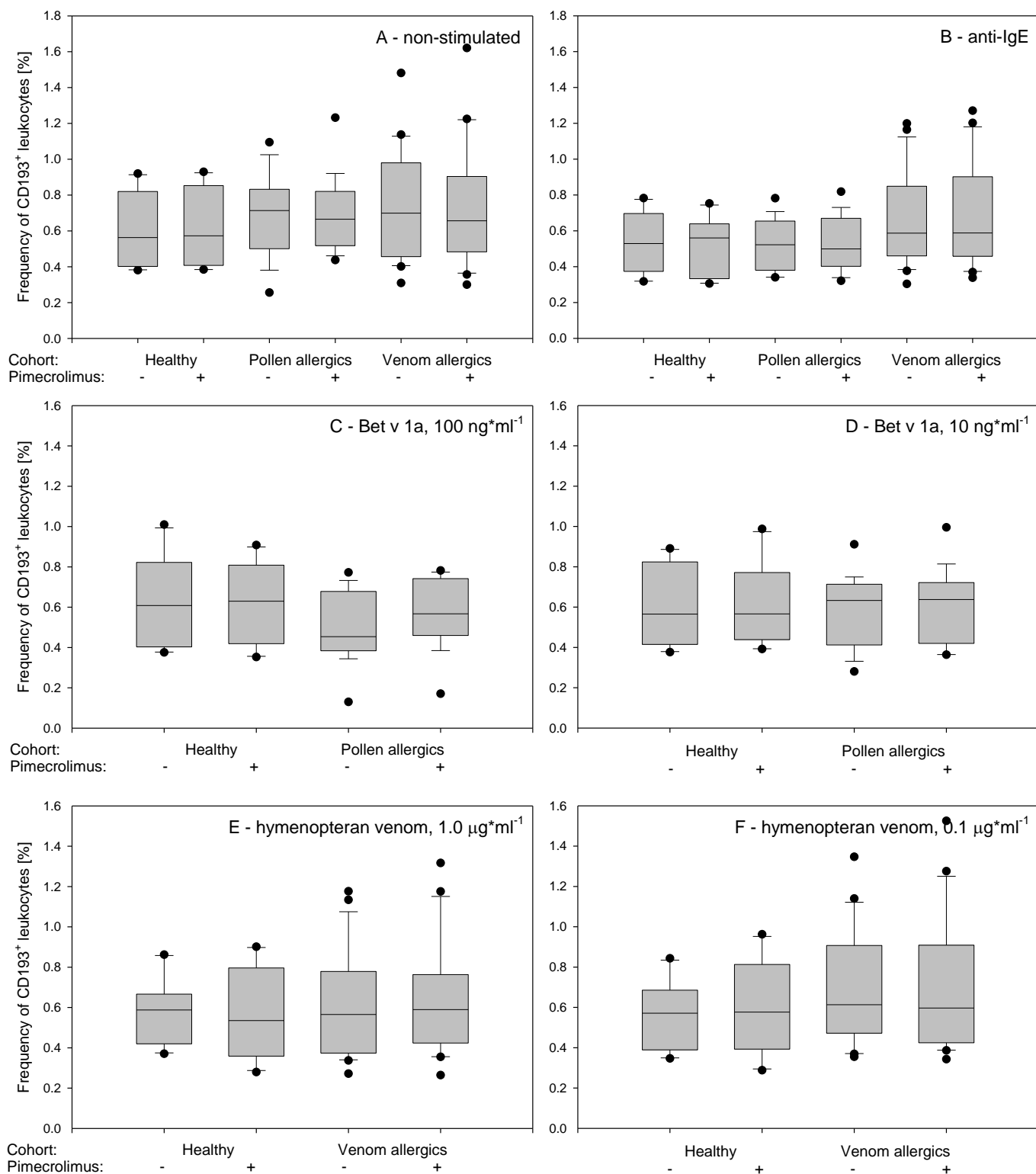

Figure S3

Supplement: S3 Fig — The frequency [%] of SSClowCD193-PE+ cells among the total leukocytes is shown. The IL-3-primed basophils were pre-treated or not with 2.5 μMol pimecrolimus and analyzed prior to (A) and after activation with anti-IgE (B), birch pollen allergen Bet v 1a at 100 ng*ml-1 (C) and 10 ng*ml-1 (D), and hymenopteran venom at 1.0 μg*ml-1 (E) and 0.1 μg*ml-1 (F). The Tukey box plots are shown, the mean values are presented as lines, and the 5th and 95th percentiles are displayed as symbols. (PDF) [file pone.0142953.s003.pdf]

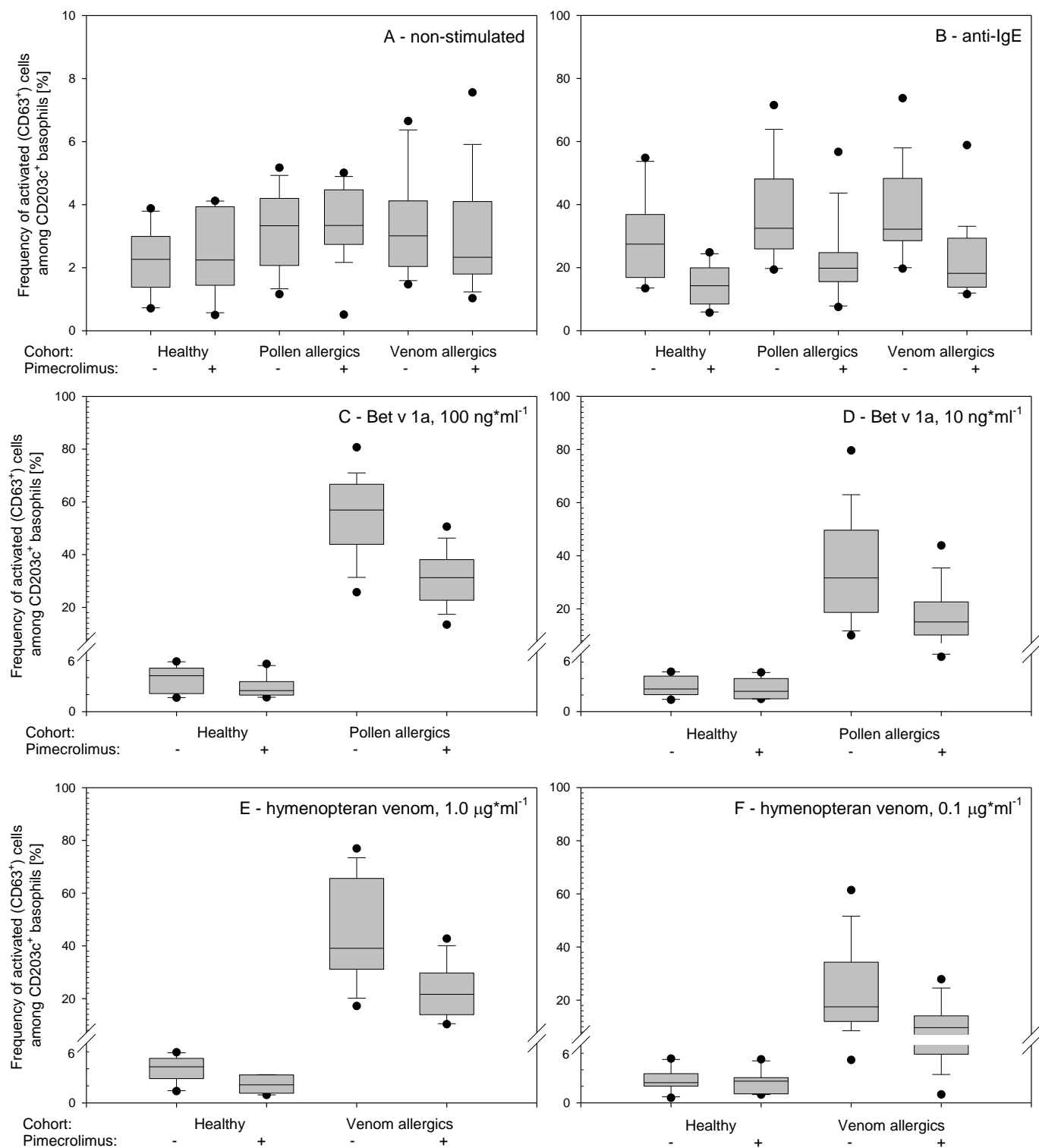

Figure S4

Supplement: S4 Fig — The frequency [%] of CD63-FITC+ cells among the total basophils (gated as SSClowCD203c-PE+) is shown. The IL-3-primed basophils were pre-treated or not with 2.5 μMol pimecrolimus and analyzed prior to (A) and after activation with anti-IgE (B), birch pollen allergen Bet v 1a at 100 ng*ml-1 (C) and 10 ng*ml-1 (D), and hymenopteran venom at 1.0 μg*ml-1 (E) and 0.1 μg*ml-1 (F). The Tukey box plots are shown, the mean values are presented as lines, and the 5th and 95th percentiles are displayed as symbols. (PDF) [file pone.0142953.s004.pdf]

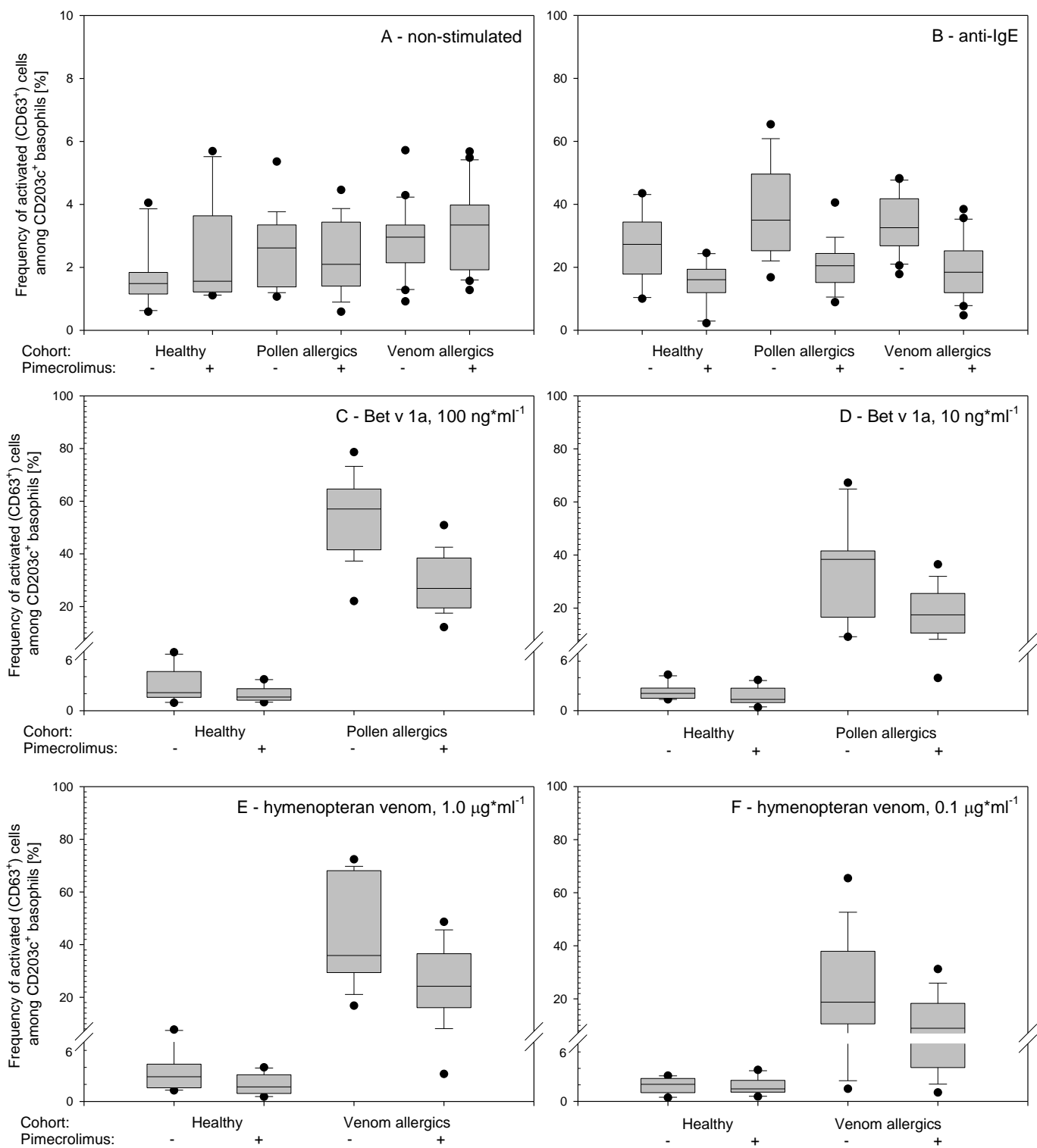

Figure S5

Supplement: S5 Fig — The frequency [%] of CD63-PerCP+ cells among the total basophils (gated as SSClowCD203c-APC+) is shown. The IL-3-primed basophils were pre-treated or not with 2.5 μMol pimecrolimus and analyzed prior to (A) and after activation with anti-IgE (B), birch pollen allergen Bet v 1a at 100 ng*ml-1 (C) and 10 ng*ml-1 (D), and hymenopteran venom at 1.0 μg*ml-1 (E) and 0.1 μg*ml-1 (F). The Tukey box plots are shown, the mean values are presented as lines, and the 5th and 95th percentiles are displayed as symbols. (PDF) [file pone.0142953.s005.pdf]

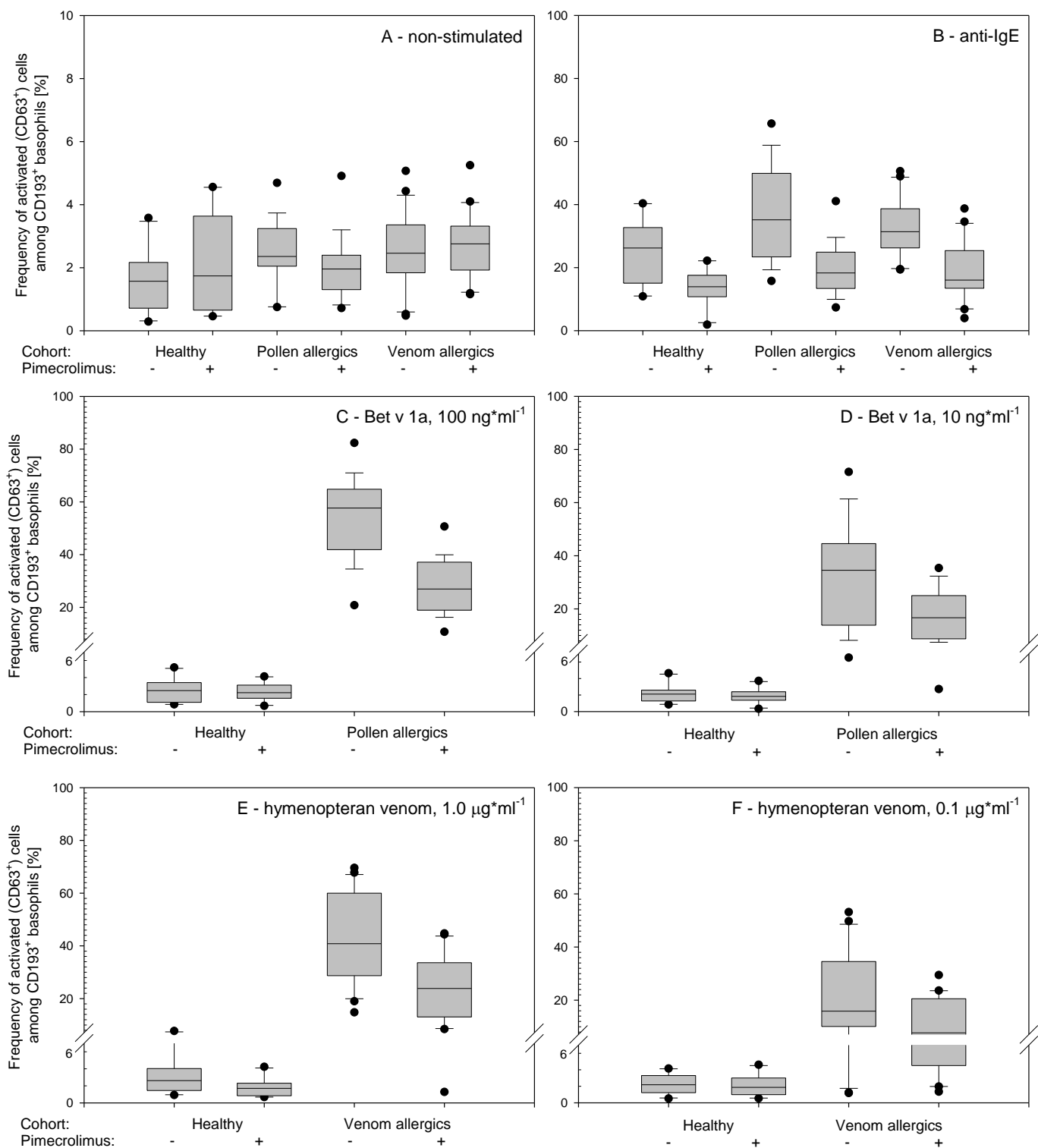

Figure S6

Supplement: S6 Fig — The frequency [%] of CD63-PerCP+ cells among the total basophils (gated as SSClowCD193-PE+) is shown. The IL-3-primed basophils were pre-treated or not with 2.5 μMol pimecrolimus and analyzed prior to (A) and after activation with anti-IgE (B), birch pollen allergen Bet v 1a at 100 ng*ml-1 (C) and 10 ng*ml-1 (D), and hymenopteran venom at 1.0 μg*ml-1 (E) and 0.1 μg*ml-1 (F). The Tukey box plots are shown, the mean values are presented as lines, and the 5th and 95th percentiles are displayed as symbols. (PDF) [file pone.0142953.s006.pdf]

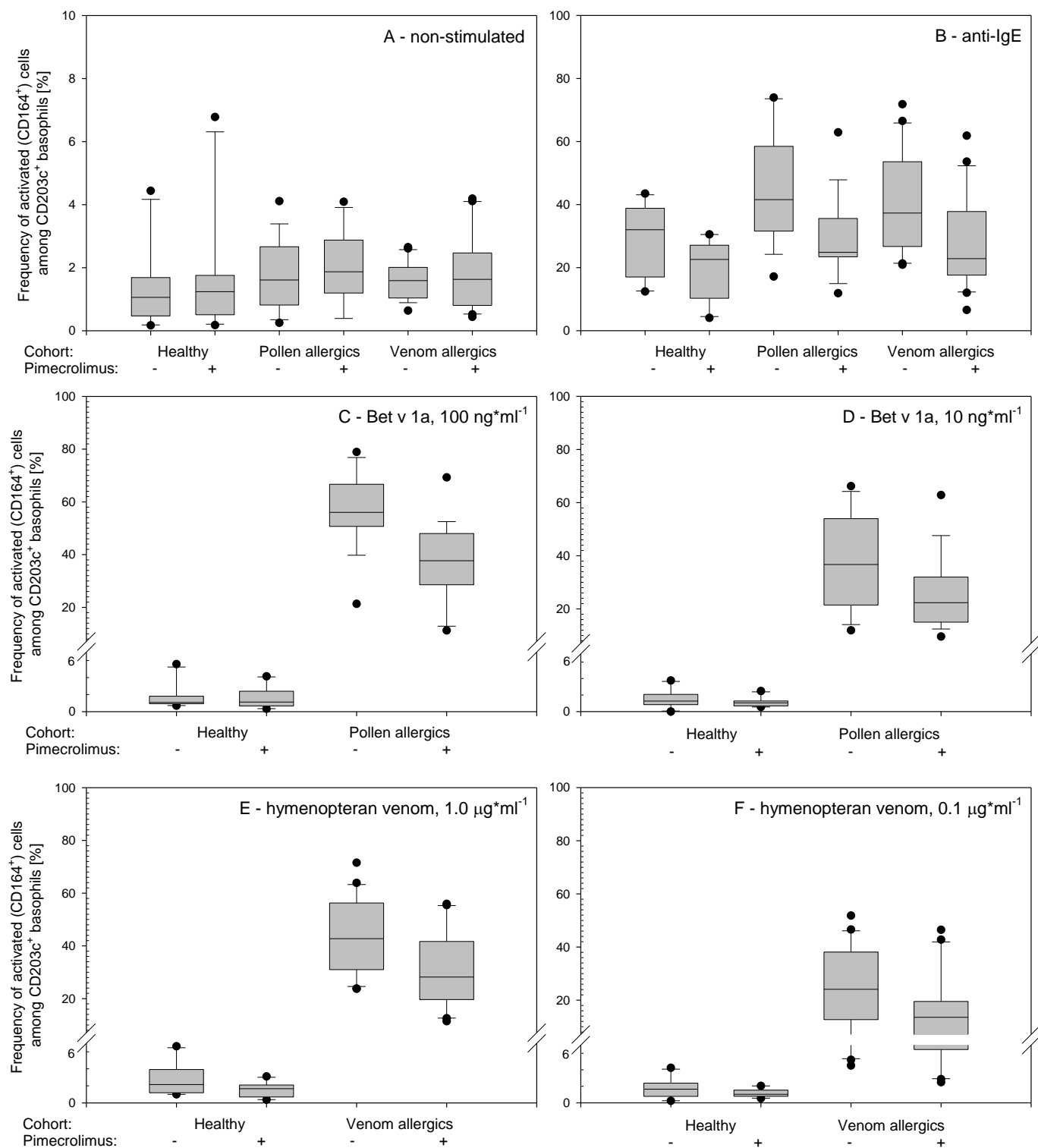

Figure S7

Supplement: S7 Fig — The frequency [%] of CD164-FITC+ cells among the total basophils (gated as SSClowCD203c-APC+) is shown. The IL-3-primed basophils were pre-treated or not with 2.5 μMol pimecrolimus and analyzed prior to (A) and after activation with anti-IgE (B), birch pollen allergen Bet v 1a at 100 ng*ml-1 (C) and 10 ng*ml-1 (D), and hymenopteran venom at 1.0 μg*ml-1 (E) and 0.1 μg*ml-1 (F). The Tukey box plots are shown, the mean values are presented as lines, and the 5th and 95th percentiles are displayed as symbols. (PDF) [file pone.0142953.s007.pdf]

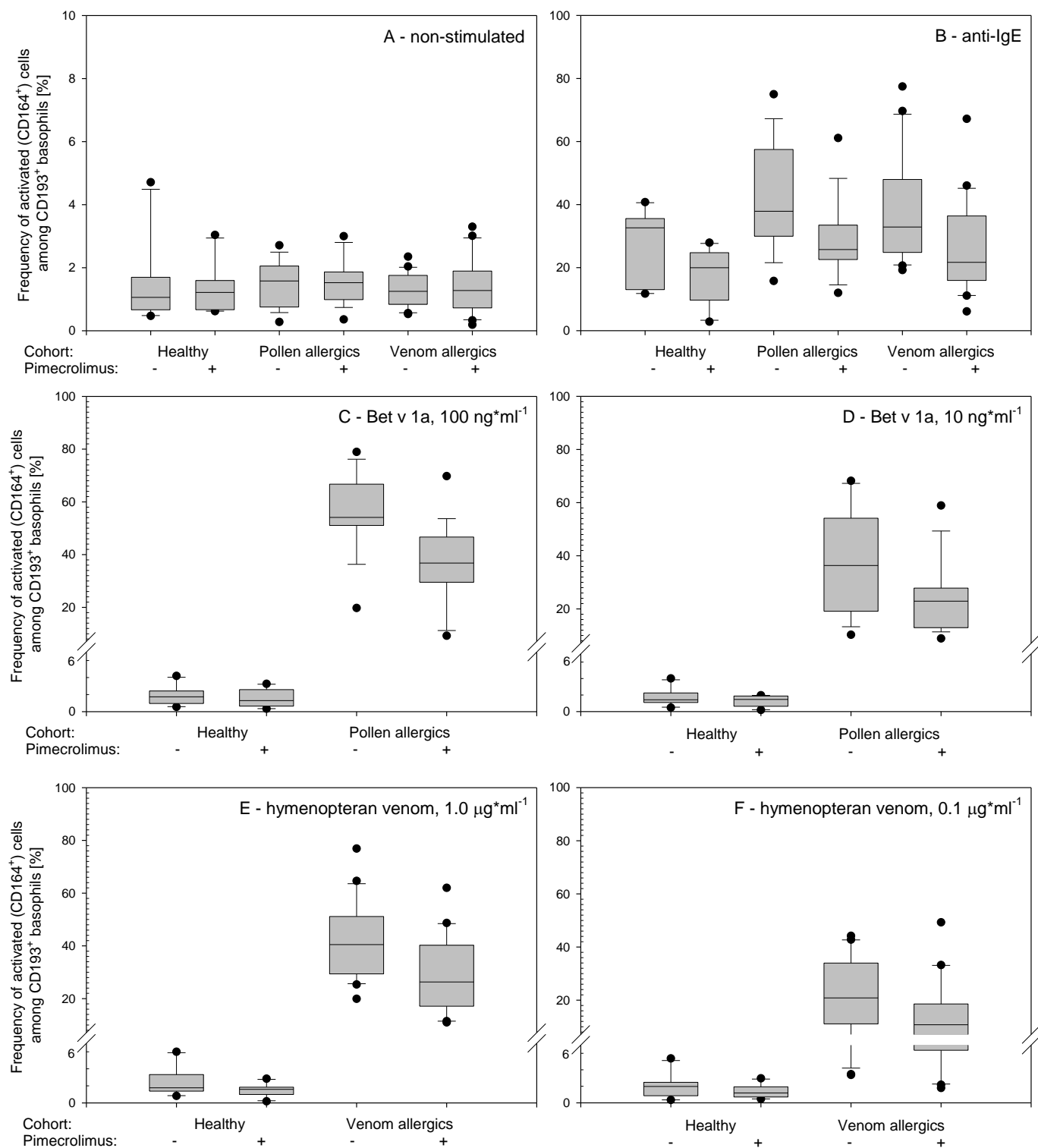

Figure S8

Supplement: S8 Fig — The frequency [%] of CD164-FITC+ cells among the total basophils (gated as SSClowCD193-PE+) is shown. The IL-3-primed basophils were pre-treated or not with 2.5 μMol pimecrolimus and analyzed prior to (A) and after activation with anti-IgE (B), birch pollen allergen Bet v 1a at 100 ng*ml-1 (C) and 10 ng*ml-1 (D), and hymenopteran venom at 1.0 μg*ml-1 (E) and 0.1 μg*ml-1 (F). The Tukey box plots are shown, the mean values are presented as lines, and the 5th and 95th percentiles are displayed as symbols. (PDF) [file pone.0142953.s008.pdf]
